# Engineered patterns of Notch ligands Jag1 and Dll4 elicit differential spatial control of endothelial sprouting

**DOI:** 10.1016/j.isci.2022.104306

**Published:** 2022-04-27

**Authors:** Laura A. Tiemeijer, Tommaso Ristori, Oscar M.J. A. Stassen, Jaakko J. Ahlberg, Jonne J.J. de Bijl, Christopher S. Chen, Katie Bentley, Carlijn V.C. Bouten, Cecilia M. Sahlgren

**Affiliations:** 1 Faculty for Science and Engineering, Biosciences, Åbo Akademi University, Turku, 20500, Finland; 2 Department of Biomedical Engineering, Eindhoven University of Technology, Eindhoven, 5612 AZ, the Netherlands; 3 Institute for Complex Molecular Systems (ICMS), Eindhoven University of Technology, Eindhoven, 5612 AZ, the Netherlands; 4 The Biological Design Center and Department of Biomedical Engineering, Boston University, Boston, MA 02215, USA; 5 Turku Bioscience Centre, Åbo Akademi University and University of Turku, Turku, 20500, Finland; 6 The Wyss Institute for Biologically Inspired Engineering, Harvard University, Boston, MA 02115, USA; 7 The Francis Crick Institute, London, NW1 1AT, UK; 8 Department of Informatics, King’s College London, London, WC2B 4BG, UK

**Keywords:** Bioengineering, Biological sciences, Developmental biology, Tissue engineering

## Abstract

Spatial regulation of angiogenesis is important for the generation of functional engineered vasculature in regenerative medicine. The Notch ligands Jag1 and Dll4 show distinct expression patterns in endothelial cells and, respectively, promote and inhibit endothelial sprouting. Therefore, patterns of Notch ligands may be utilized to spatially control sprouting, but their potential and the underlying mechanisms of action are unclear. Here, we coupled *in vitro* and *in silico* models to analyze the ability of micropatterned Jag1 and Dll4 ligands to spatially control endothelial sprouting. Dll4 patterns, but not Jag1 patterns, elicited spatial control. Computational simulations of the underlying signaling dynamics suggest that different timing of Notch activation by Jag1 and Dll4 underlie their distinct ability to spatially control sprouting. Hence, Dll4 patterns efficiently direct the sprouts, whereas longer exposure to Jag1 patterns is required to achieve spatial control. These insights in sprouting regulation offer therapeutic handles for spatial regulation of angiogenesis.

## Highlights

• Dll4-functionalized patterns guide direction and location of angiogenic sprouts
• Jag1-functionalized patterns do not have relevant effects on angiogenic sprouts
• Modeling predicts that differences are due to distinct signaling temporal dynamics

## Graphical abstract

**Figure:**
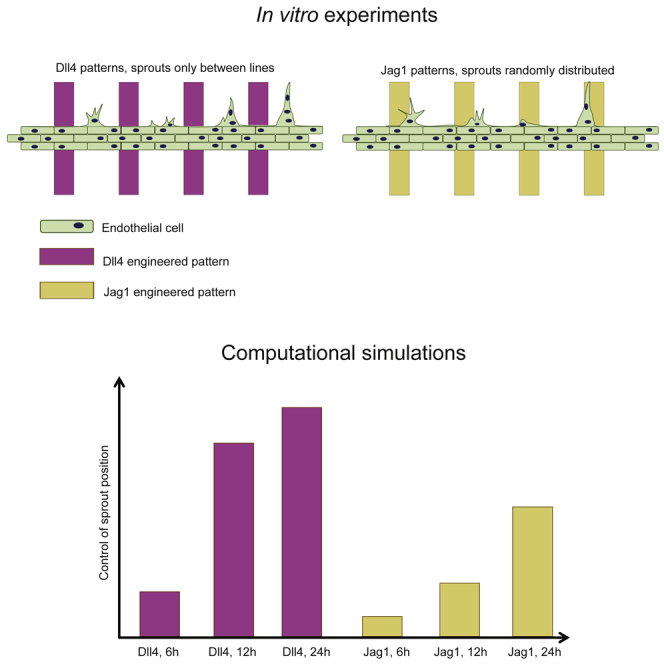

## Introduction

The formation of new blood vessels through angiogenesis is a combination of processes, starting with an endothelial sprout and ending with vascular tube maturation and vessel quiescence (Potente and Carmeliet, 2017). These processes need to be spatially guided to ensure the establishment of a functional vasculature. Proper vascularization of tissues (Carmeliet, 2005) is imperative for regenerative medicine and tissue engineering (TE) (Jain et al., 2005; Rouwkema and Khademhosseini, 2016; Kant and Coulombe, 2018).

The Notch signaling pathway is a key regulator of angiogenesis (Holderfield and Hughes, 2008; Phng and Gerhardt, 2009) and may offer a therapeutic handle for spatial control of endothelial sprouting. Genetic removal of Notch ligands and receptors results in disorganized and nonfunctional vasculature and embryonic lethality (Xue et al., 1999; Krebs et al., 2000; Gale et al., 2004; Limbourg et al., 2005). In addition, deregulated Notch activity is linked to pathological angiogenesis (Kofler et al., 2011; Lobov and Mikhailova, 2018). The Notch ligands Delta-like ligand 4 (Dll4) and Jagged1 (Jag1) have distinct roles during angiogenesis (Benedito et al., 2009; Marchetto et al., 2020). Dll4- and Jag1-mediated activation of the receptor Notch1 regulates tip cell versus stalk cell selection and the formation of new branching points during sprouting angiogenesis. This regulation occurs via a crosstalk with Vascular Endothelial Growth Factor (VEGF) signaling, a key driver of angiogenesis (Holderfield and Hughes, 2008; Phng and Gerhardt, 2009; Potente et al., 2011; Blanco and Gerhardt, 2013), and includes Dll4-mediated lateral inhibition. VEGF-VEGFR2 signaling induces Dll4 expression in endothelial cells (Hellström et al., 2007; Phng and Gerhardt, 2009) and the adoption of a migratory tip cell phenotype. Dll4-mediated Notch1 activation in neighboring cells reduces their expression of VEGFR2, causing a lateral inhibition of the tip cell phenotype in these cells, which are thus induced to retain a stalk cell phenotype (Hellström et al., 2007; Lobov et al., 2007; Sainson and Harris, 2008; Benedito et al., 2009). Therefore, the crosstalk between VEGF and Dll4-mediated Notch signaling enhances the differentiation between two adjacent tip and stalk cells. Jag1 competes with Dll4 for Notch binding, thereby counteracting the Dll4-mediated lateral inhibition process and indirectly promoting the tip cell phenotype (Benedito et al., 2009; Marchetto et al., 2020). This Dll4-Jag1 competition needs to be tightly balanced to obtain physiological angiogenesis, characterized by a characteristic pattern of tip cells alternated by stalk cells. This underlies a dose-dependent effect of the ligands. For example, although deletion of Jag1 decreases sprouting, also excessive and unbalanced expression of Jag1 can counteract the formation of the characteristic tip-and-stalk pattern and lead to pathological sprouting (Kang et al., 2019). How this competition between ligands is balanced and how ligand-receptor signaling specificity is achieved is under intense investigation (Benedito et al., 2009; Kakuda and Haltiwanger, 2017; Luca et al., 2017). Despite numerous studies on the role of Notch in angiogenesis, how the spatial organization of Jag1 and Dll4 ligands affect endothelial sprouting still needs to be elucidated.

External patterns of these ligands may provide novel strategies to spatially control and direct endothelial sprouting for regenerative medicine and TE. Several *in vitro* techniques have been proposed to engineer organized vascular networks (Raghavan et al., 2010; Kim et al., 2013; Moya et al., 2013; Malheiro et al., 2016; Rouwkema and Khademhosseini, 2016; Kant and Coulombe, 2018). These methods mainly rely on bioengineering approaches such as PDMS scaffolds, 3D (bio)printing, and microchips, which provide the structural layout of the microvasculature *ex vivo*, but have limited potential to integrate with native or engineered tissues. Direct targeting and stimulation of angiogenesis *in vivo* and *in vitro* have been extensively investigated; examples include injectable biomaterials enriched with Notch signaling components and VEGF (Cao et al., 2010), 3D collagen and Matrigel gels incorporating Notch ligands (Antfolk et al., 2017; van Engeland et al., 2019), and decoy-based targeting of Notch ligands (Kangsamaksin et al., 2015). Although these approaches effectively induce growth of the vasculature, they do not exert control over the location and direction of the blood vessels. We believe that spatially controlled initiation of endothelial sprouting may be an essential first step toward the engineering of functional (micro)vasculature.

Computational models simulating the crosstalk between VEGF and Notch signaling have contributed to our understanding of angiogenesis. Simulations from agent-based models have resulted in a wide range of experimentally validated predictions of sprouting angiogenesis, highlighting the strongly dynamic nature of the tip-stalk phenotypic competition (Bentley et al., 2008; Bentley et al., 2009, 2014; Jakobsson et al., 2010; Ubezio et al., 2016; Zakirov et al., 2021) and the importance of the temporal dynamics of Dll4-Notch1 signaling in defining vascular density (Bentley and Chakravartula, 2017). The role of Jag1 in the VEGF-Notch crosstalk was computationally investigated by Boareto et al. (2015b), who obtained simulations in agreement with previous *in vivo* experiments with Notch1-Jag1 signaling dysregulation (Benedito et al., 2009). Further simulations based on this model could thus help to elucidate the impact of ligand organization on the spatial control of angiogenesis.

**Figure 1:**
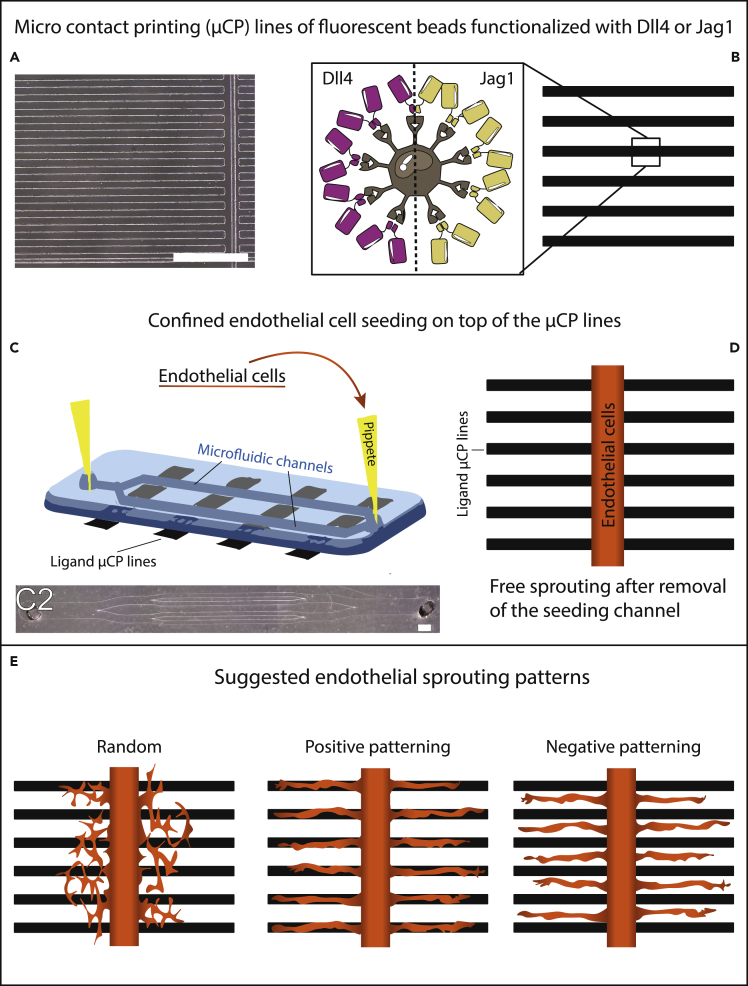
Schematic overview of experimental approach To compare possible different influences of Dll4 vs Jag1 patterns on endothelial sprouting, the method previously described in Tiemeijer et al. (2018) was adapted. (A–C and E) In short, microcontact printing (μCP) stamps (A) were inked with fluorescent beads functionalized with either Dll4 or Jagged1, and lines of 100 μm with 100 μm spacing were printed on glass slides (B). (D) In addition, endothelial cells were seeded on top, via microfluidic channels (C and C2) perpendicular to the lines. After channel removal, Matrigel was added, and the endothelial cells were left to sprout freely for 24 h. With respect to the ligand-functionalized lines, we envision the possibility of three different spatial sprouting patterns (E), random, positive, and negative patterning. Scale bar represents 1mm.

Here, we compared the potential of patterns of external Dll4 and Jag1 to spatially control the location and direction of endothelial sprouts (Figure 1). Dll4 micropatterned lines directed the sprout initiation and location in between the lines. These data are in agreement with our previous results, demonstrating that spatial patterns of externally presented Dll4 ligands inhibit angiogenic sprouting (Tiemeijer et al., 2018). Interestingly, spatial patterns of external Jag1 had no evident impact on the location of sprouts. We adapted a previous *in silico* model (Boareto et al., 2015b) to simulate our experimental setting and elucidate the mechanisms behind the ligand-specific effects on angiogenic patterning. The simulations suggest that the lower spatial control of Jag1 compared with Dll4 derives from different timing of Notch activation, which results from the different affinity of Notch1 to the distinct ligands (Luca et al., 2017). The data show that Dll4 is a more potent spatial regulator of endothelial sprouting compared with Jagged1, and its unique ability to exert spatial control could be exploited as a tool for vascular patterning and engineering.

## Results

### A tailored *in vitro* system enabled a controlled comparison of the effect of Notch ligands on endothelial sprouting

**Figure 2:**
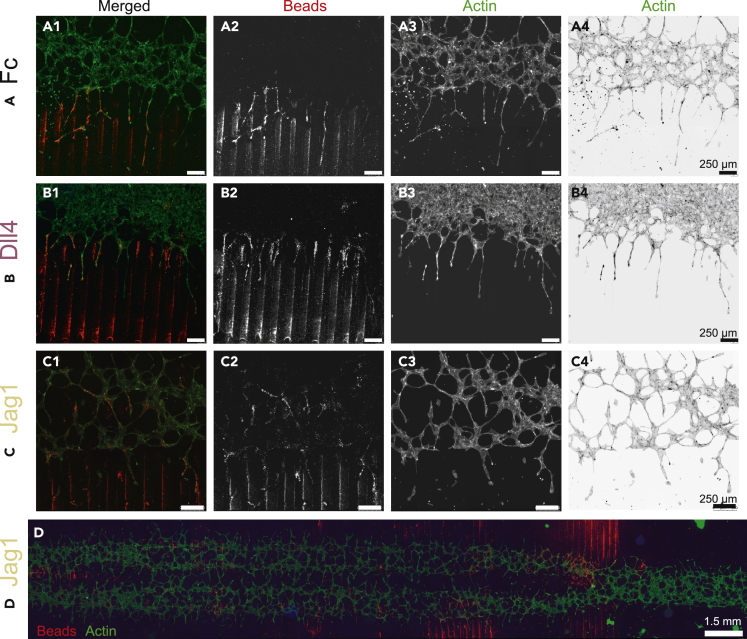
Endothelial sprouting on substrates with different ligand lines after 24 h (A–D) The top rows (A–C) show zoomed-in sections of representative samples with Jag1, Dll4, and Fc lines, highlighting the fluorescent beads (red) used in the μCP ink (column 2), and the actin skeleton (green) of the endothelial cells, shown as normal and inverted for clearer visual assessment (column 3 and 4, respectively). The bottom row (D) shows a representative image of a whole Jag1 sample, from which ROIs were taken for analysis. As expected, Fc lines did not seem to affect the sprouts, and sprouting did seem to be confined to space between Dll4 lines. However, Jag1 lines did not seem to affect endothelial sprouts, and a similar crossing behavior as in Fc samples could be observed. Scale bars in A–C represent 250 μm. Scale bar in D represents approximately 1.5 mm.

The effect of Jag1 and Dll4 patterns in spatially controlling endothelial sprouting was investigated in a tightly controlled platform that we previously developed (Tiemeijer et al., 2018) (Figure 1). Briefly, microcontact printing (μCP) was adopted to print 100-μm wide fluorescent lines, functionalized with either Jag1 or Dll4, alternated with 100 μm spacing, on glass slides (Figures 1A and 1B). Line functionalization with Fc fragments was used as a control. Microfluidic channels placed on top of the micropatterned lines enabled spatially confined seeding of human umbilical vein endothelial cells (HUVECs) in 150-μm and 300-μm wide lines perpendicular to the ligand patterns (Figures 1C and 1D). This confined seeding was important to control and study the initiation of sprouting from a restricted area, to mimic sprouting from a native vessel *in vivo*, here represented by a confined endothelial monolayer *in vitro*. Immediately after cell adherence, the channels were removed. After channel removal, Matrigel was placed on top of the cells and the patterned substrate, to allow free cellular sprouting for a period of 24 h, after which cells were fixated for analysis. At the onset of sprouting, the endothelial cells were in contact with the ligand-functionalized lines beneath them, which was expected to influence their tip-stalk cell fate. Moreover, the path of the sprouts during sprouting could be expected to be influenced by ligand-functionalized lines encountered at the sprouting front. To determine the effects of the lines on the sprout location and direction (Figure 1E), fluorescent microcopy was adopted to visualize the actin cytoskeleton, the cell nuclei, and the fluorescent beads in the functionalized lines (Figure 2). As expected, Fc lines did not appear to affect the sprouts (Figures 2A1–2A4), and some sprouts crossed multiple lines. A similar crossing behavior could be observed for Jag1 samples (Figures 2C1–2C4), which exhibited endothelial cells on the lines, in their near vicinity, and in the space between lines. Therefore, visual assessment of the Jag1 samples did not indicate that patterning is controlled by this ligand. In contrast, in agreement with our previous findings, the sprouts in the Dll4 samples did seem to remain confined to the space between lines, consistent with negative patterning (Figures 2B1–2B4).

**Figure 3:**
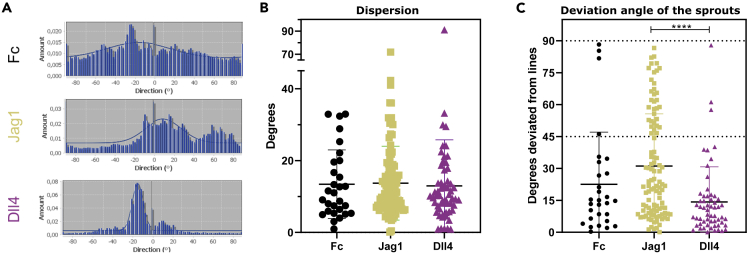
The effects of ligand patterns on sprout direction Direction of sprouts analyzed with directionality plugIn (ImageJ). (A–C) Representative histograms for sprouts on Jag1, Dll4, and Fc of selected single ROIs, normalized to the direction of the ligand lines. The dispersion of the fitted graph through the histogram for every ROI is reported in (B). The deviation angles of the sprouts with respect to the line direction, shown in (C), are significantly larger for Jag1 samples compared with Dll4 samples (p < 0.0001). See also Figure S1. Data represented as mean ± SD, N = 29/106/58 ROIs pooled from a total of three Fc/4 Jag1/4 Dll4 microchips, respectively, from all experimental rounds combined. ROIs with a goodness of a fit that resulted less than 0.2 were not considered.

**Figure 4:**
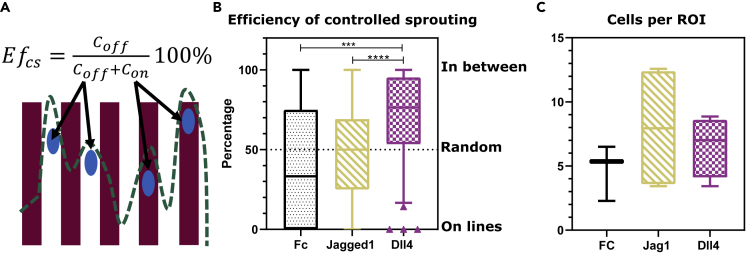
The efficiency of controlled sprouting (Ef_cs_) induced by ligand patterns (A and B) Ef_cs_ is defined by the formula in (A) and quantifies the level and type of control of the functionalized lines over the spatial location of the endothelial sprouts. Below the formula, a schematic is presented of the patterned lines (red lines), the cell nuclei (blue ellipses), and the corresponding outline of the sprouting front (discontinuous green line). The formula of Ef_cs_ then accounts for the amount of cells in the subregion of the ROI in between the ligand lines (C_off_, white area) as a percentage of the total amount of cells (C_off_ + C_on_) in that ROI. In the definition, C_on_ represents the number of cells in the subregion of the ROI on top of the ligand lines (red area). This definition of Ef_cs_ enables the identification of different sprouting patterns and control: Ef_cs_ ≈ 50% for random patterning and no control; Ef_cs_ ≈ 0% for high control toward positive patterning; and Ef_cs_ ≈ 100% for high control toward negative patterning. Comparison of the Ef_cs_ between Fc, Jag1, and Dll4 line samples is shown in (B), where boxplots represent N = 31/112/58 ROIs pooled from a total of three Fc/four Jag1/4 Dll4 microchips, respectively. There is a significant difference between the Ef_cs_ of Jag1 and Dll4 samples (p < 0.0001) and between the Fc and Dll4 (p = 0.0004). (C) No significant difference in the number of cells per ROI. In (B) and (C), data are represented as boxplots, where the boxes span from the 25^th^ until the 75^th^ percentile, the whiskers extend down to the 10^th^ percentile and up to the 90^th^ percentile, and the horizontal lines represent the median values.

### Sprouts strongly orient parallel to Dll4, but not Jag1 lines

Regions of interest (ROIs) were identified, by visual assessment, to perform image analysis. ROIs were defined as nonoverlapping regions, adjacent to the original seeding areas of the initially confined endothelial cells, where cells (1) were alive and viable; (2) displayed a sprouting phenotype; (3) were contiguous in the seeded area, mimicking a monolayer; (4) were able to sprout from the seeded area into the ROI; and (5) were exposed to visible lines of functionalized ligand.

First, we analyzed if and how the Jag1 and Dll4 functionalized lines influence the sprout orientation. For every ROI, the average orientation of the actin cytoskeleton of the cells was obtained based on fitted graphs through a direction histogram (Figure 3A). For a better comparison across samples, the orientation angles were normalized such that the ligand functionalized lines of the corresponding sample have a 0° angle. The resulting dispersions of the fitted graphs were not significantly different across samples (Figure 3B). Similarly, when orientation angles between −90° and +90° were considered (Figure S1), no significant difference across samples was observed for the average orientation angles, with angle averages around 0° in all conditions; this is consistent with the symmetry of the system with respect to the direction of the lines. To analyze whether the ligand lines induce sprouts to follow the line direction or to deviate from it, we computed the absolute value of the orientation angles, which we refer to as “deviation angle” (Figure 3C). The deviation angles of sprouts of Jag1 samples were significantly larger than those of the Dll4 samples (Figure 3C). Taken together these results indicate that, although sprouts generally orient in the direction of the lines, the sprouts of Jag1 samples are not hindered to sprout further on reaching Jag1 lines nor are dictated to follow the direction of the lines, in contrast to sprouts on Dll4-patterns.

### In contrast to Dll4, Jag1 lines cannot influence the sprout location

To further characterize the difference between Jag1 and Dll4 lines, the efficiency of controlled sprouting (Ef_cs_) was calculated (Tiemeijer et al., 2018) (Figure 4). Ef_cs_ represents the control of the functionalized lines over the spatial location of the endothelial sprouts. In particular, different sprouting patterns (Figure 1E) correspond to different Ef_cs_ values: Ef_cs_ ≈ 50% for random patterning; Ef_cs_ ≤ 50% for positive patterning; and Ef_cs_ ≥ 50% for negative patterning. Moreover, Ef_cs_ values closer to extreme percentages (0 and 100%) correspond to higher spatial control. To calculate the Ef_cs_, the ROIs were divided into two subregions with identical surface areas adjacent to each other. The subregions were located on the lines or in between the lines (On and Off). The number of cells in those areas were counted and used in the formula depicted in Figure 4A. Here, Ef_cs_ is the percentage of the number of cells that stay in between the lines (C_off_) calculated from the total number of cells in that ROI. C_on_ is the number of cells that are on the ligand-functionalized lines. The Ef_cs_ of the Jag1 samples was found to be around 50% and was not significantly different from the Ef_cs_ of the control. The Ef_cs_ of the Dll4 samples (Ef_cs_ = 70.17 ± 28.59, mean ± SD) was significantly increased compared with both control and Jag1 samples (Fc: Ef_cs_ = 42 ± 38.57 and Jag1: Ef_cs_ = 47.77 ± 30.69, mean ± SD) (Figure 4B). The Ef_cs_ was computed based on multiple nuclei per ROI. To ensure that the difference in Ef_cs_ did not result from a difference in cell number per ROI per sample group, the number of cells per ROI in the samples was analyzed; no significant difference was observed (Figure 4C). Overall, these data show that Dll4 lines are more potent than Jag1 lines in dictating the sprout location.

### Simulations qualitatively mimic cell fate differences on different lines

**Figure 5:**
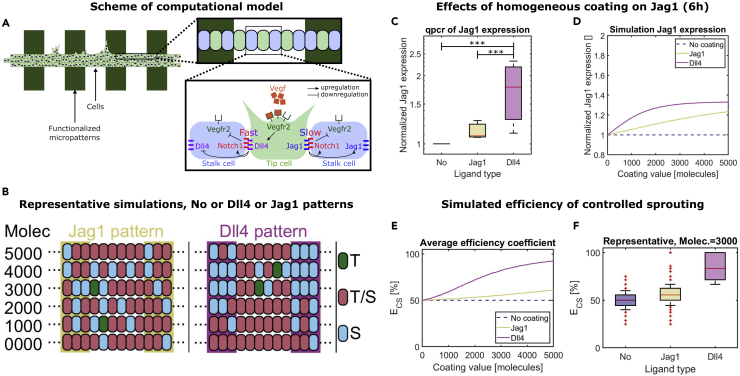
The computational model quantitatively replicates the experiments (A) Due to periodicity, cell-cell signaling was modeled only for a small portion of the cells on the patterned substrate. The first enlargement shows the cells that were analyzed in the simulations. The second enlargement shows the main features of the simulated signaling network. (B) Representative computational results, with each row corresponding to one simulation, with different ligand concentrations on the micropatterns (decreasing from top to bottom). Without ligands on the patterns (bottom row) most cells have a tip/stalk (T/S) hybrid phenotype (red). With increasing concentration, Dll4 induces the stalk (S, blue) phenotype for cells on top of the lines, whereas Jag1 does not have evident effects. Only few tip (T, green) cells were observed. (C and D) The effects of homogeneous coating of Fc, Jag1, or Dll4 on the gene expression of Jag1 were compared between experimental (C) and computational (D) data for the time period of 6 h. qPCR revealed a significant difference of Jag1 expression for Dll4 versus Fc samples (p < 0.0001) and for Dll4 versus Jag1 samples (p < 0.001). For the computational data, averages over 10,000 simulations for each concentration are reported. (E) The efficiency of controlled sprouting was computed for simulations of cells on patterned lines with varying ligand densities. The graph reports the averages of the computed values over 10,000 simulations. (F) Boxplots obtained for the simulations with D_line_ = J_line_ = 3000 molecules. The simulations captured the higher spatial control of Dll4 lines, compared with Jag1 lines, on the location of the sprouts (E and F). In (C) and (F), data are represented as boxplots, where the boxes span from the 25th until the 75th percentile, the whiskers extend down to the 10th percentile and up to the 90th percentile, and the horizontal lines represent the median values.

To investigate the underlying mechanisms determining the differential behavior of endothelial cells exposed to Dll4 and Jag1 lines, we simulated our experiments by adapting a previous mathematical model of Notch cross-talking with VEGF that considers Dll4- and Jag1-mediated signaling (Boareto et al., 2015a). Briefly, the model simulates signaling among neighboring cells by assuming that both Dll4 and Jag1 lead to Notch activation, with faster Dll4-Notch1 binding rate and activation compared with Jag1-Notch1. This assumption is motivated by the higher affinity of Dll4 to Notch1, which leads to a higher probability of Dll4-mediated Notch1 activation, compared with Jag1-mediated activation, in a shorter time (Luca et al., 2017). The model also assumes that Notch activation downregulates VEGFR. This signaling crosstalk leads to three possible phenotypes based on the level of VEGFR activity: migratory tip cells with very high activity; proliferative stalk cells with very low activity; and slowly migrating tip/stalk hybrid cells, with moderate activity. To simulate our experiments, taking advantage of the spatial periodicity of the system, we considered cell signaling among cells on a limited area of the substrate, corresponding to cells located in between (half of) two patterned lines (Figure 5A). The Notch receptors of the cells on top of the lines were assumed to bind and be activated by the Notch ligands on the patterns in the same way as Notch ligands of neighboring cells. A more detailed model and parameter description can be found in the supplemental information.

Cell-cell signaling was simulated among 12 adjacent endothelial cells for 12 h, in accordance and within the time frame of the experiments. Representative images of the computational results (Figure 5B) show that most cells had a hybrid tip/stalk phenotype when not presented to Notch functionalized ligands, corresponding to randomly distributed (slowly) migrating cells (Figure 5B, bottom row). With increasing Notch ligand concentrations, Dll4 patterns increasingly forced the cells on top of the lines to obtain a stalk phenotype (Figure 5B right), in agreement with experiments where the tip cells were observed only in between the lines (Figure 4B). Jag1, on the other hand, did not have any evident effect on the phenotypes (Figure 5B left), also corresponding to the experimental results (Figure 4B).

### Simulations quantitatively mimic the experiments in terms of tip cell distribution

To better compare the experiments and simulations, we calibrated the model parameters D_line_ and J_line_, corresponding to the Notch ligand content coated on top of the lines. For calibration, via qPCR, we obtained the Jag1 expression of HUVECs exposed to homogeneously coated ligands for 6 h (Figure 5C), and we simulated these experiments by considering different ligand densities (Figure 5D). The computational results mimicked the higher Jag1 expression response elicited by Dll4 coating, compared with Jag1 coating (Figures 5C and 5D). As a result of this comparison, we chose D_line_ = J_line_ = 3000 molecules as a representative ligand content for the simulations, considering this as a parameter value leading to a very high Dll4 response balanced by relatively low Jag1 effects.

After calibration, a quantitative comparison between sprouting experiments and simulations was obtained by plotting the boxplots of Ef_cs_ values resulting from 10,000 simulations, performed with the identified representative ligand concentration. The model could quantitatively mimic the experiments (Figure 5F): Dll4 ligands induced Ef_cs_ values around the value 100%, corresponding to sprouts only in between the lines; Jag1 elicited a random sprout distribution, very similar to the Fc control, corresponding to Ef_cs_ values around the value 50%. Further simulations (Figure 5E) indicated that increasing the Dll4 concentration on the patterns causes an increasing distribution of tip cells in between the lines, and stalk cells on top of the lines, whereas Jag1 has a much lower effect even at very high concentrations. Overall, these simulations indicate that the experimentally observed response of cells to the lines can be replicated with a computational model assuming that Dll4-Notch1 activation occurs at a higher rate than the Jag1-Notch1 counterpart, highlighting a possible role of the signaling temporal dynamics.

### Parameter exploration confirms that Notch signaling temporal dynamics is a key factor

**Figure 6:**
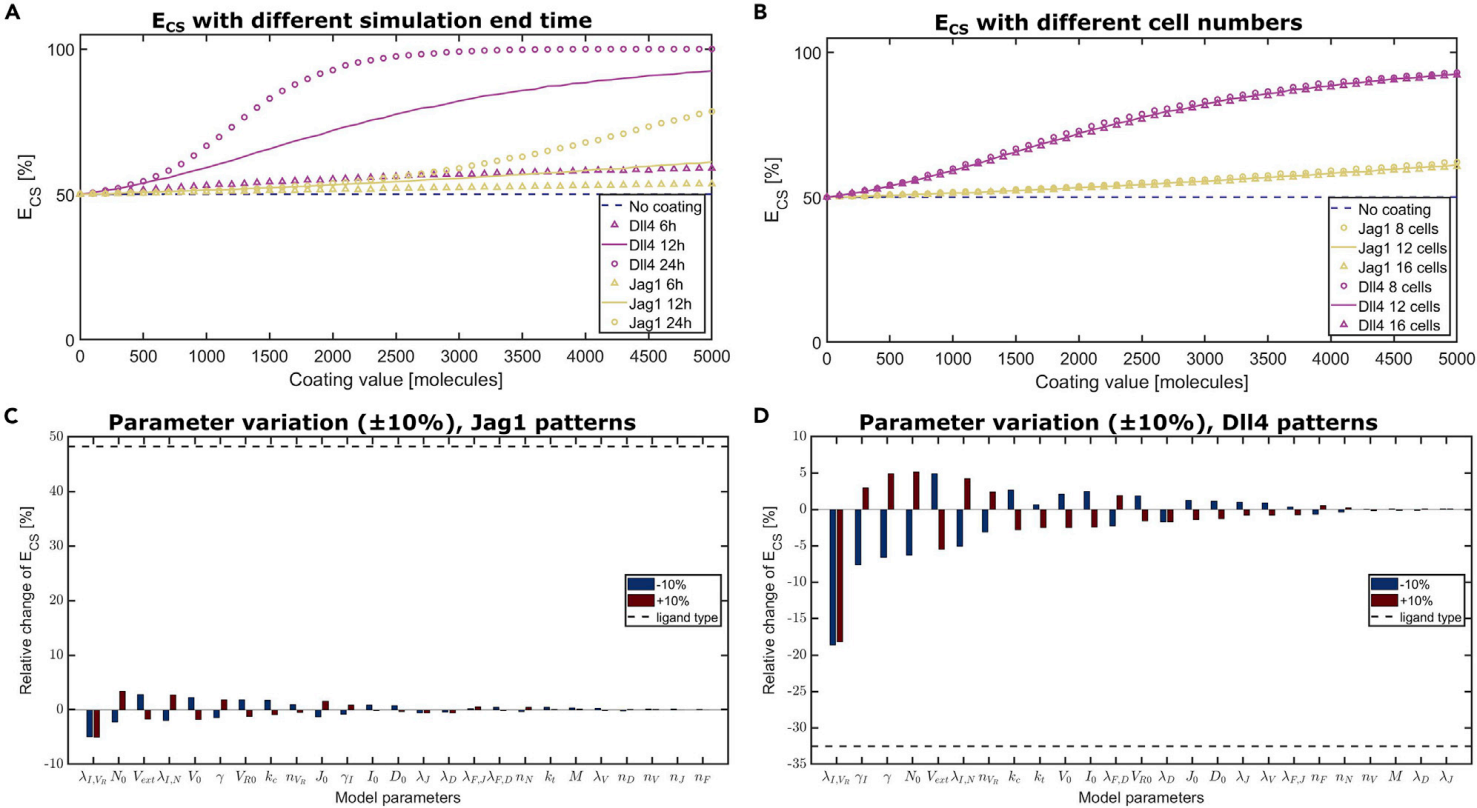
Parameter exploration identifies duration of ligand induction as a major determinant of efficiency of control (A and B) Efficiency of controlled sprouting computed for simulations with varying ligand content on the patterns, for different end time of the simulations (A), and different number of cells on the patterns (B). (C and D) Sensitivity of the efficiency of controlled spatial sprouting computed for Jag1 patterns (C) and Dll4 patterns (D) to 10% variations of the parameters describing the signaling dynamics, compared with switching the ligand type (dashed lines). In all cases, the values reported are averages over 10,000 simulations obtained by varying the initial protein content in each cell.

After verifying that the model can replicate the experiments, we performed additional simulations to confirm the importance of the signaling temporal dynamics compared with other parameters. To this aim, we performed a parameter exploration by varying the final time of the simulations between 6 and 24 h (Figure 6A), the number of cells on the lines (Figure 6B) and the other model parameters describing the cell signaling dynamics (Figures 6C and 6D). Interestingly, Figure 6A shows that the timing of cell migration away from the original configuration is a major determinant of the effects of Dll4 and Jag1. Generally, the effect of the ligand functionalized lines positively correlates with the time necessary for cells to leave the patterned lines; a longer time spent on the patterns gives a stronger effect. The simulations suggest that Jag1 might have a higher effect if the cells stay on top of the patterns for a longer period, so as to allow a longer time for Jag1 to activate Notch1. Overall, variations of the other model parameters (Figures 6B–6D) did not cause large variations of the results, especially compared with the variations caused by switching the ligand type (Dll4 to Jag1 and vice-versa, Figures 6C and 6D). In conclusion, the simulations indicate that the choice of ligand and the temporal dynamics are major determinants of the endothelial sprouting response of cells to the functionalized lines.

## Discussion

A spatially organized vasculature is important for tissue functionality, and spatially controlling angiogenesis is fundamental for regenerative medicine and TE applications (Jain et al., 2005; Rouwkema and Khademhosseini, 2016; Kant and Coulombe, 2018). Current approaches do not enable optimal control over the location and direction of dynamic sprouts. We believe that spatially controlling the initiation of endothelial sprouting is key for applying further control over angiogenesis in an engineered vasculature context. Notch signaling is a key regulator of sprouting angiogenesis (Holderfield and Hughes, 2008; Phng and Gerhardt, 2009). Engineering micropatterns of Notch ligands might thus enable spatial control of sprouts. Here, by combining *in vitro* and *in silico* models, we investigated the potential and underlying mechanisms of Jag1 and Dll4 lines controlling the location and direction of endothelial sprouts. In agreement with our previous findings (Tiemeijer et al., 2018), we confirmed the potency of Dll4 lines for negative patterning of endothelial sprouting (Figure 1E). Our results indicate that spatially controlled external patterns of Dll4 can overrule the native Dll4-mediated tip-stalk cell selection and dictate the location of stalk cells. Despite the opposite role of Jag1 in native angiogenesis (Benedito et al., 2009; Marchetto et al., 2020), Jag1 lines did not elicit positive patterning but rather random patterning (Figure 1E). Specifically, Jag1 lines had a very low efficiency to control the sprout location (Figure 4) and a higher deviation angle between the lines and the sprout orientation (Figure 3). Dll4 is therefore much more potent than Jag1 in spatially controlling sprouting. Our simulations indicate that this difference arises from the distinct affinity of the ligands to Notch1 (Luca et al., 2017).

The lower affinity of Jag1 to Notch1, compared with Dll4, causes a slower rate of Jag1-mediated Notch1 activation (Kakuda and Haltiwanger, 2017); this is a key assumption of the computational model and enabled the simulations to capture the experimental results (Figure 5). In the simulations, the μCP Dll4 rapidly activates the Notch1 receptor of cells on Dll4 lines, thereby forcing them to the stalk cell phenotype and allowing sprout formation only in between the patterns. In contrast, Jag1 requires a longer time to activate Notch1 because of its lower affinity (Luca et al., 2017). Cells can therefore sprout on Jag1 lines before being forced to a stalk phenotype. Previous studies have highlighted the temporal dynamics of Notch as a key factor for native angiogenesis (Bentley and Chakravartula, 2017). Our findings provide a different angle, emphasizing the importance of the receptor-ligand binding and activation rate for the spatial control of angiogenesis with Notch micropatterns. In particular, our results suggest that a higher spatial control of endothelial sprouts could be achieved by controlling the time of ligand exposure (Figure 6A) or by engineering ligands with higher affinity to Notch1.

To model signaling among cells, the original assumptions of the model of Boareto et al. (2015b) were adopted, including the assumption that Dll4 and Jag1 have different affinity to Notch1 because of Fringe activity. Fringes are enzymes that glycosylate the Notch extracellular domain (Brückner et al., 2000); as a result of their activity, Dll4- and Jag1-mediated activation of Notch1 increases or decreases, respectively (Benedito et al., 2009). Recent studies indicate that Dll4 and Jag1 have different affinity irrespective of Fringe (Luca et al., 2017). The computational model could be modified to account for this, but our conclusions would not change: Jag1 patterns would require more time to activate Notch1 compared with Dll4. This time difference arises from affinity and probability. As reported by Luca et al. (Luca et al., 2017), “the probability that a Notch1 receptor is engaged with a ligand during the period of cellular tension application to the receptor is higher if the affinity is higher.” In other words, higher affinity translates into a higher probability of Notch1 activation and, thus, a larger Notch1 activation in a limited amount of time; this can be rephrased by saying that having a higher affinity among ligands and receptors is equivalent to having a higher rate of Notch1 activation as mediated by such ligand-receptor interaction. In principle, at least in the computational model, the lower rate of Jag1-mediated Notch1 activation might be compensated by a higher concentration of Jag1 on the patterns: this would increase the probability of successful binding and Notch1 activation in a fixed time and therefore increase the activation rate. However, it is unclear whether this high concentration of Jag1 could lead to physiological sprouting, due to the dose-dependent effect of this ligand in the context of angiogenesis. In fact, as indicated by a recent study (Kang et al., 2019) combining a 3D *in vitro* model of angiogenesis and an extension of the model adapted in the present study (Boareto et al., 2015b), excessive concentrations of Jag1 can result in antiangiogenic responses (Kang et al., 2019). Therefore, future applications should still focus on Dll4 patterns rather than Jag1 patterns.

Our findings can be translated toward controlling angiogenesis for TE and regenerative medicine. Spatially controlling the initiation of endothelial sprouting can be a central step toward providing a controlled functional vasculature. Here, Dll4 ligands were clearly more potent than Jag1 in dictating the location of endothelial sprouts. Therefore, future studies in this context should choose Dll4 rather than Jag1, although the use of Dll4 might present challenges in 3D. In 2D, we were able to confine the sprouts in between two Dll4 lines because of the inhibitory effect of this ligand. However, cells have increased freedom in 3D, which makes their confinement and guidance more challenging. Dll4 functionalized materials might nevertheless be used in 3D to avoid cells from sprouting through specific homogeneously functionalized surfaces, to avoid sprouting toward some areas.

In conclusion, by combining *in vitro* and *in silico* models, we demonstrated that engineering patterns of Jag1 have a lower potential to control the location and direction of endothelial sprouts than Dll4 patterns. The simulations indicate that this difference arises from the lower affinity of Jag1 to Notch1 compared with Dll4, which results in a slower Jag1-mediated activation of Notch1. To achieve a higher spatial control of endothelial sprouting, future studies could aim at engineering Notch ligands with higher affinity to Notch1.

### Limitations of the study

The present study presents some limitations, which were minimized to enhance the accuracy of the data interpretation. The experimental method involves manual steps that could result in small sample imperfections. To drastically limit their possible influence on the data analysis, the criteria for the ROI selection were set very strictly. In addition, in contrast to native angiogenesis, our *in vitro* and *in silico* models do not expose cells to VEGF gradients; their inclusion would tremendously increase the complexity of the *in vitro* system design and validation. In particular, it would require a more controlled addition of VEGF within microchips, similar to Zheng et al. (Zheng et al., 2017); however, combining these devices with the micropatterning of Notch ligands would be extremely challenging. Computationally, the effects of VEGF gradients could be investigated by extending the computational model with filopodia formation (Venkatraman et al., 2016), cell shape changes, and movement (Vega et al., 2020). These mechanisms, affected by VEGF gradients (Gerhardt et al., 2003), were not considered here because we focused on the initial response of cells leaving the original seeding area. The absence of these factors may explain why the computational model did not predict alternating tip-stalk phenotypes for cells on control substrates (Figure 5B), in contrast to previous predictions for native angiogenesis (Boareto et al., 2015b). In absence of VEGF gradients, filopodia formation, and of cell shape changes, several feedback loops in the VEGF-Notch crosstalk are necessary to establish the tip-stalk pattern at the gene level (Bentley and Chakravartula, 2017). Each loop has a period of around 4 to 6 h (Ubezio et al., 2016). Therefore, in agreement with experiments, our simulation end time of 12 h is too short for cells to establish the characteristic tip-stalk pattern. Choosing 12 h is here justified by the short time necessary for cells to leave the initial seeding position in our devices, much shorter than both 24 h (our experiment end time) and the asymptotic conditions simulated for native angiogenesis (Boareto et al., 2015b).

## STAR★Methods

### Key resources table

**Table:** 

REAGENT or RESOURCE	SOURCE	IDENTIFIER
**Chemicals, peptides, and recombinant proteins**		

active human Dll4 protein fragment	Abcam, HEK293 cells	ab108557; lot#:GR3172950-14
recombinant human Jagged1/fc chimera	R&D systems	Cat#1277-JG; Lot#RZL2219031
ChromePure human IgG Fc fragment	Jackson Immuno Research	Code: 0009-000-008; Lot#135334
protein G fluorescent particles	Spherotech	Cat#PGFP-0562-5
SYLGARD^TM^ 184 Silicone Elastomer Kit (PDMS)	DOW	GMID: 01672921; CAS: 100-41-4
Matrigel membrane basis growth factor reduced	Corning	Cat#354230; Lot#8190005
Gelatin	Sigma, porcine	CAS: 900708,
Endothelial cell base media 2 and supplement pack endothelial cell GM2	Promocell	Cat#C-39211; Lot#454M232
Su-8 Masters, made with Su-8 2000 photoresist, Su-8-2050 and Su-8 Developer	Tiemeijer et al. 2018 master and design, MicroChem (chemicals)	www.microchem.com
Phalloidin Atto-488 Bioreagent	Merck Life Science NV	N/A
DAPI	Merck Life Science NV	CAS: 28718-90-3
HOT FIREPol® EvaGreen® qPCR Mix Plus (ROX)	Solis Biodyne	08-24-00008
Revertaid Reverse Transcriptase	Thermo fisher	EP0441
Protein G	Pierce	77675
Recombinant dll4-fc	R&D Systems	10185-D4
Recombinant jag1-fc	R&D Systems	1277-JG
Recombinant human IgG1-Fc	R&D Systems	110-HG-100

**Critical commercial assays**		

Nucleospin Kit	Marcherey-Nagel	740955.5

**Deposited data**		

Fluorescence microscopy, image analysis, quantitative PCR data, computational data	This paper	https://doi.org/10.4121/19235784

**Experimental models: Cell lines**		

Human: Human Umbilical Vein Endothelial Cells	Lonza	Cat#C2519A; Lot#0000704189

**Oligonucleotides**		

Jag1 Primer: Forward: AATGGCTACCGGTGTGTCTG; Reverse: CCCATGGTGATGCAAGGTCT	Sigma-Aldrich	N/A

**Software and algorithms**		

Fiji (Image J)	Schindelin et al. (Schindelin et al., 2012)	https://imagej.net/software/fiji/
GraphPad Prism 8.0.2	GraphPad	https://www.graphpad.com/scientific-software/prism/
LAS X small 3.4.2	Leica microsystems	https://www.leica-microsystems.com/products/microscope-software/p/leica-las-x-ls/
Original code	This paper	https://doi.org/10.4121/19235793

**Other**		

Corona Discharge Generator	Tantec HF	N/A

### Resource availability

#### Lead contact

Further information and requests for resources and reagents should be directed to and will be fulfilled by the lead contact, Cecilia Sahlgren (cecilia.sahlgren@abo.fi).

#### Materials availability

This study did not generate new unique reagents.

### Experimental model and subject details

#### Primary cells source

HUVECs were purchased from Lonza. Product code: C2519A, Lot number: 0000704189. Tissue Acquisition number: P826. Pooled Donors. Sex: MALE/FEMALE MIXED. Race: B,B,A,C,C,C.

### Method details

#### Experimental design (objectives and design of study)

In this study, the effects of Jag1 and Dll4 micropatterned lines on endothelial sprouting were compared. Fc fragments were used a control. We looked at the effects on sprout location and direction with fluorescent microscopy. Briefly, 6 different experimental rounds were performed; each round on a different day. Per round, approximately 20 million Human Umbilical Vein Endothelial Cells (HUVECs) from one passage number (P3, P4, or P6) were distributed among 8 microchips, corresponding to 2 Fc, 4 Jag1, and 2 Dll4 chips/samples per round. Thus, the experiment was performed for a total of 48 chips (12 Fc, 24 Jag1, 12 Dll4). 22 chips that showed signs of channel leakage, failed μCP or cell death during or at the end of the experimental period (24h) were omitted from further analysis. *In silico* modeling was adopted to investigate if the experiments could be explained by the effects of the Notch ligand patterns on the dynamics of cell signaling.

#### Fabrication of the soft lithography masters

As described in our previous study (Tiemeijer et al., 2018), standard soft lithography was used to create SU-8 masters for both μCP stamps and cell seeding channels. The same SU-8 masters were used in this study. Briefly, Su-8 2050 was spun at 500 rpm for 10 s, followed by 30 s at 2000 rpm, prebaked 5 min at 65°C and 15 min at 95°C. UV exposure was performed with a mask containing the desired features for 35 s at 13mNcm−2. Post baking 5 min at 65°C and 8 min at 95°C and finally developed for an hour with SU-8 developer.

#### Fabrication of μCP stamps and cell seeding channels

PDMS consisting of 10:1 w/v base to curing agent (Sylgard 184) was mixed, degassed by centrifuging and poured over the corresponding master before final removal of air bubbles in vacuum. After curing at 65°C overnight, the PDMS was taken of the masters and cut into stamps with dimensions of 100 μm lines spaced 100 μm and channels with dimensions of 150 μm and 300 μm wide, spaced by 300 or 500 μm, with 1.5 mm inlets and outlets (Figures 1A and 1C2).

#### Cell culture

HUVECs (Lonza, pooled, Cat#: C2519A Lot#: 0000704189) were cultured in Endothelial Cell Base Media 2 (Promocell) with addition of the supplement pack endothelial cell GM2 (Promocell) with final concentrations of 0.02 ml/ml Fetal Calf Serum (FCS), 5 ng/ml human recombinant Epidermal Growth Factor (hEGF), 10 ng/ml human recombinant basic Fibroblast Growth Factor (hbFGF), 20 ng/ml human recombinant Insulin-like Growth Factor (R3-IGF-1), 0.5 ng/ml human recombinant Vascular Endothelial Growth Factor 165 (VEGF), 1 μg/ml ascorbic acid, 22.5 μg/ml heparin, and 0.2 μg/ml hydrocortisone and was supplemented with additional 1% penicillin/streptomycin. Cell culture flasks were coated with 0.1% gelatin (Porcine, Sigma) before culture in 37°C, 5% CO_2._ Cells with passage numbers of 3-6 were used for the experiments.

#### μCP of ligand functionalized lines

Similar to our previous study (Tiemeijer et al., 2018), 50 μl (10 μg/ml) of active human Dll4 protein fragment (Abcam) or 4.3 μl (200 μg/ml) of recombinant human Jagged1/fc chimera (R&D systems) or 0.2 μl (2.2 mg/ml) ChromePure human IgG Fc fragment (Jackson Immuno Research) was incubated with 100 μl (0.1% w/v) protein G fluorescent particles (Spherotech, purple, 0.4–0.6 μm) overnight at 5°C, resulting in μCP ink with a maximum of 0.5 μg of Dll4 ligand immobilized to the beads per sample or molar% equivalents of Jag1 and Fc fragments. Before inking of the μCP stamps with ligand-bead mixture, the stamps were made hydrophilic by treatment with atmospheric plasma for 9 s (Corona Discharge Generator, Tantec HF). μCP stamps and ink were incubated for 30 min at RT and dried using N_2_ gas prior to printing on glass slides, which were sterilized with 70% ethanol and additional UV light treatment (5 min). The μCP stamps were left to adhere to the glass for 30 min at RT before removal.

#### Confined cell seeding and sprouting assay

Microfluidic channels were placed on the printed glass slides orientated perpendicular to the ligand functionalized lines and gently pressed to contact the substrate and seal the microchannels. P200 pipette tips (without filter) were placed in the inlets of the channels as fluidic reservoirs that allowed feeding of the system by gravity (Figure 1C). The channels were coated with 1% gelatin in PBS, pipetted in one of the tips, for 5 min at 37°C, 5% CO2, followed by careful flushing out of the gelatin solution with pre-warmed media (18 h at 37°C, 5% CO_2_). HUVECs in pre-warmed media (5∗10^6^ cells/ml) were seeded directly via the pipette tips and were left to adhere for approx. 2.5 h at 37°C, 5% CO_2_ monitoring against cell aggregates and ensuring sufficient flow through the channels. After cell adherence the channels were cautiously taken off the glass slides, and Matrigel®matrix (15% in total amount of media, growth factor reduced, Corning; lotnr. 00034014) was placed on top of the HUVECs, after setting shortly followed by remaining media. The HUVECs were left to sprout unconfined for 24 h in 37°C, 5% CO_2_.

#### Fluorescent microscopy and image analysis

The cell samples were fixated for 30 min at RT with 3.7% formaldehyde in PBS. The fixed samples were permeabilized with 0.5% triton X-100 in PBS for 15 min. PBS was used as washing buffer during staining with the markers Phalloidin (Atto488) for the actin cytoskeleton and DAPI for the nuclei respectively. Tile scan acquisition was done using a Leica DMi8 TIRF microscope. Regions of interest (ROIs) were defined as mentioned in the results and identified by visual assessment. Direction analysis for every ROI was performed with the Fourier components method of the Directionality plugin in Fiji (Image J). ROIs with a “goodness of a fit” value lower than 0.2 were excluded from subsequent analysis, as the fitting graph was judged inaccurate for those cases. The average orientation and the dispersion were extracted from the graphs fitted on the histograms. The average direction was normalized to the direction of ligand functionalized lines. Sprouts that deviate to the left from the line direction would correspond to a positive direction and sprouts that deviate to the right would correspond to a negative direction. The efficiency of patterning was quantified similar to our previous study (Tiemeijer et al., 2018), by using an equation adjusted from Frimat et al. (Frimat et al., 2009):

(Equation 1) $$Ef_{cs}=\frac{C_{off}}{C_{off}+C_{on}}100\%$$

where Ef_cs_ is the efficiency of controlled sprouting of the endothelial cells in between the lines in percentage, C_off_ the number of cells in the region A of the ROI (in between the lines) and Con the number of cells in the region B of the ROI (on top of the lines), where A and B have equal dimensions and surface, and together are one ROI. The cells were counted using the Cell Counter plugin in Fiji (Image J). Statistical analysis was done using non-parametric ANOVA (Kruskal-Wallis) with Dunn’s multiple comparisons test.

#### qPCR

Notch response after 6-h ligand induction was tested on HUVECs cultured in wells coated with protein-G and either 25 nM recombinant Fc-Dll4, 25 nM recombinant Fc-Jag1 or 25 nM Fc. RNA was isolated using a Nucleospin kit (Macherey-Nagel) and 100–150 ng RNA was converted to cDNA by Revertaid Reverse Transcriptase (Thermo Fischer). qPCR was run on a QuantStudio3 qPCR machine (Applied Biosystems), with as mastermix Hot FirePol EvaGreen plus ROX (Solis Biodyne). Primer of Jag1 consisted of; Forward: AATGGCTACCGGTGTGTCTG, Reverse: CCCATGGTGATGCAAGGTCT.

#### Computational model

A previous computational model (Boareto et al., 2015b) was adopted to simulate cell-cell signaling occurring among the cells in our experiments. The model was adapted to simulate a row of endothelial cells with periodic boundary conditions (with the first cell connected to the last cell in the row), with boundaries corresponding to half of two consecutive lines patterned with ligands (Figure 5A). Half of the cells were considered in contact with functionalized ligands on the substrate. The model relies on a system of ordinary differential equations describing the time variation of the Notch and VEGF signaling proteins within each cell. Briefly, external VEGF is assumed to bind and activate VEGFR, which leads to Dll4 upregulation (Blanco and Gerhardt, 2013). Dll4 can then bind to Notch1 in both the same cell, leading to cis-inhibition (Sprinzak et al., 2010), or neighboring cells, causing transactivation. Notch cis-inhibition and transactivation can also be mediated by the other Notch ligand, Jag1. Notch activation leads to an increase in Notch intracellular domain, which in turn causes downregulation of Dll4 (Shimojo et al., 2011) and VEGFR (Blanco and Gerhardt, 2013), and upregulation of Jag1 and Notch1, in the receiving cell (Manderfield et al., 2012). Finally, the Notch intracellular domain is assumed to activate Fringe (Morales et al., 2002), which in turn influences the (cis- and trans-) binding rate of Notch1 with its ligands. This increases the Dll4-Notch1 binding rate and decreases the Jag1-Notch1 binding rate (Kopan and Ilagan, 2009).

The modelling assumptions translate into a system of ordinary differential equations describing the dynamics of VEGF and Notch signaling in each cell. In particular, the equations focus on the time variations of free Notch (*N*_*i*_), Dll4 (*D*_*i*_), Jag1 (*J*_*i*_), Notch intracellular domain (*I*_*i*_), VEGFR (*V*_*R*,*i*_), and activated VEGFR (*V*_*i*_), for each cell with index *i*∈*N*. The variation over time of these proteins is described by the following system of ordinary differential equations:

(Equation 2) $$\frac{dN_{i}}{dt}=N_{0}H^{S}\left(I_{i},\;\lambda_{I,N},n_{N}\right)-N_{i}\left[\left(k_{C}D_{i}+k_{T}D_{i,ext}\right)H^{S}\left(I_{i},\;\lambda_{F,D},n_{F}\right)+\left(k_{C}J_{i}+k_{T}J_{i,ext}\right)H^{S}\left(I_{i},\;\lambda_{F,J},n_{F}\right)\right]-\gamma N_{i}$$

(Equation 3) $$\frac{dD_{i}}{dt}=D_{0}H^{S}\left(I_{i},\;\lambda_{I,D},n_{D}\right)H^{S}\left(V_{i},\;\lambda_{V,D},n_{D}\right)-D_{i}\left[k_{C}H^{S}\left(I_{i},\;\lambda_{F,D},n_{F}\right)N_{i}+k_{T}N_{i,ext}\right]-\gamma D_{i}$$

(Equation 4) $$\frac{dJ_{i}}{dt}=J_{0}H^{S}\left(I_{i},\;\lambda_{I,J},n_{J}\right)-J_{i}\left[k_{C}H^{S}\left(I_{i},\;\lambda_{F,J},n_{F}\right)N_{i}+k_{T}N_{i,ext}\right]-\gamma J_{i}$$

(Equation 5) $$\frac{dI_{i}}{dt}=k_{T}N_{i}\left[D_{i,ext}H^{S}\left(I_{i},\;\lambda_{F,D},n_{F}\right)+J_{i,ext}H^{S}\left(I_{i},\;\lambda_{F,J},n_{F}\right)\right]-\gamma_{S}I_{i}$$

(Equation 6) $$\frac{dV_{R,i}}{dt}=V_{R0}H^{S}\left(I_{i},\;\lambda_{I,V_{R}},n_{V_{R}}\right)-k_{T}V_{R,i}V_{ext}-\gamma V_{R,i}$$

(Equation 7) $$\frac{dV_{i}}{dt}=k_{T}V_{R,i}V_{ext}-\gamma_{S}V_{i}$$

Here, *N*_0_, *D*_0_, *J*_0_, *V*_*R*0_ represent the production rate of Notch1, Dll4, Jag1, and VEGFR, respectively. *γ* is the degradation rate of Notch ligands, VEGF ligands, and inactive receptors; *γ*_*S*_ indicates the degradation rate of activated Notch and VEGFR. *k*_*C*_ and *k*_*T*_ label the rate of cis-inhibition and transactivation of Notch receptors by Notch ligands when no Fringe is expressed. *k*_*T*_ also represents the rate of activation of VEGFR by external VEGF (indicated with the model parameter *V*_*ext*_). The rate of protein production, cis-inhibition, and transactivation varies over time as influenced by Notch and VEGFR activation, as captured by the shifted Hill function $H^{S}\left(X,\;\lambda_{X,Y},n\right)=\lambda_{X,Y}+(1-\lambda_{X,Y})/(1+{\left(X/X_{0}\right)}^{n})$. *X* and *Y* are here auxiliary labels to indicate that *H*^*S*^ describes the variation in production of *Y* due to variations in the activation of *X* with respect to a reference value *X*_0_. *n* represents the sensitivity of the production rate to these variations. Finally, *λ*_*X*,*Y*_ describes the effect of these variations on *Y*: *λ*_*X*,*Y*_ = 1 has no effects; *λ*_*X*,*Y*_<1 corresponds to downregulation; and *λ*_*X*,*Y*_>1 corresponds to upregulation. Similarly, $H^{S}\left(I,\;\lambda_{F,D},n_{F}\right)$ and $H^{S}\left(I,\;\lambda_{F,J},n_{F}\right)$ represent the production rate of the enzyme Fringe and, indirectly, the influence of Fringe on the binding and activation rate of Notch1 to Dll4 (with *λ*_*F*,*D*_) and Jag1 (with *λ*_*F*,*J*_), respectively. Finally, in the present study we define *N*_*i*,*ext*_, *D*_*i*,*ext*_, and *J*_*i*,*ext*_ as the summation of Notch1, Dll4, and Jag1 content on the patterned lines and on neighboring cells, such that:

(Equation 8) $$N_{i,ext}=\left(N_{i-1}+N_{i+1}\right)/2$$

(Equation 9) $$D_{i,ext}=\left\{ \begin{array}{cc} \left(D_{i-1}+D_{i+1}\right)/2+D_{line} & cell\;i\;is\;on\;a\;Dll4\;line\\ \left(D_{i-1}+D_{i+1}\right)/2 & otherwise \end{array}\right.$$

(Equation 10) $$J_{i,ext}=\left\{ \begin{array}{cc} \left(J_{i-1}+J_{i+1}\right)/2+J_{line} & cell\;i\;is\;on\;a\;Jag1\;line\\ \left(J_{i-1}+J_{i+1}\right)/2 & otherwise \end{array}\right.$$

The indices *i*−1 and *i*+1 correspond to the direct neighbors of the cells. As in Boareto et al. (2015a, 2015b), we therefore assumed that the rate of Notch transactivation is proportional to the average content of Notch proteins in their neighbors. In the present study, we added the parameters *D*_*line*_ and *J*_*line*_ to consider the Dll4 and Jag1 concentrations coated on the ligand-functionalized lines, thereby assuming that the Notch ligands printed on the patterns bind and activate the Notch receptors present in the cells on top of the patterned lines. The parameters *D*_*line*_ and *J*_*line*_ were calibrated via a comparison between simulations and experiments of cells on homogeneous coatings (see Figures 5C and 5D). The remaining parameter values were chosen in agreement with Boareto et al. (2015a, 2015b) and they are reported in Table S1.

Due to the periodicity of the system, only cells within a limited portion of the substrate were simulated, corresponding to the cells in between the middle portion of two adjacent patterned lines (Figure 5A). Periodic boundary conditions were assigned to cells at the edges of this area, such that the first cell was considered in contact and signaling with the last cell. Practically, if we are simulating *M* ∈*N* cells, in Equations 8, 9, and 10 the value of the function mod *M* applied to *i*−1 and *i*+1 is taken for the cells with indices 1 and *M*, respectively. This is performed to ensure that cell 1 is a neighbor of the cells with indices 2 and *M*, while cell *M* is a neighbor of the cells with indices 1 and *M*−1. The system of equations was solved with an explicit scheme, with a time-step *dt* = 0.01 h. The simulations were run with a limited end time *t*_*fin*_ equal to 12 h, unless stated otherwise. Finally, a phenotype was assigned to each endothelial cell according to the final VEGFR activation, represented by the value *V*_*i*_(*t*_*fin*_): if 0 < *V*_*i*_(*t*_*fin*_) < 100, a stalk (S) cell phenotype was assigned; 100 ≤ *V*_*i*_(*t*_*fin*_)≤300 corresponded to a hybrid tip/stalk (T/S) phenotype; and cells with *V*_*i*_(*t*_*fin*_) > 300 corresponded to tip cells (T). For the calculation of Ef_cs_, we considered that: cells predicted as tip cells and tip/stalk cells correspond to sprouting migrating cells; cells predicted as stalk cells correspond to non-sprouting cells. Ef_cs_ was computed via Equation 1, by counting the tip and tip/stalk hybrid cells that were localized on top (C_on_) or off (C_off_) the patterns.

The solutions at the limited time point of 12 h depend on the initial conditions. To account for that, all simulations were repeated 10000 times, with random initial conditions chosen such that 0 < *N*_*i*_(0), *D*_*i*_(0), *J*_*i*_(0), *V*_*R*,*i*_(0)<6000 molecules, and 0 < *I*_*i*_(0), *V*_*i*_(0)<600 molecules for all indices *i*. Averages and boxplots of the simulated Jag1 expression and Ef_cs_ were computed over the 10000 simulation runs. Random initial conditions are justified because, in the *in vitro* experiments, HUVECs were exposed to VEGF already during the initial cell culture step, before the microchip seeding. As a result of VEGF exposure, cell-cell signaling, and random cell movement, we can assume that cells in the culture flasks exhibit a random mix of tip, stalk, and hybrid phenotypes. This supports our choice of random phenotypes and protein contents as initial conditions for the microchip simulations, similar to studies with analogous models (Boareto et al., 2015a, 2015b; Loerakker et al., 2018; Ristori et al., 2020). Even changing this assumption by choosing specific ratios of phenotypes as initial conditions would not significantly change the computational results. Irrespective of the initial conditions, when strong effects from the patterns are absent, cells obtain an approximately homogeneous hybrid phenotype (Figure 5B). This general tendency towards a homogeneous hybrid phenotype would therefore cancel out any chosen distribution of cell phenotypes as initial condition and only strong and relatively fast effects from the patterns would force cells into the stalk phenotype (as in Figure 5B).

### Quantification and statistical analysis

#### Experimental results

After fluorescent imaging, multiple ROIs per chip could be potentially identified. Due to the stringent criteria for ROI selection, successful ROI identification was limited to a total of 3 Fc, 4 Jag1, 4 Dll4 chips from 4 experimental rounds. For statistical analysis, data from the selected ROIs were pooled per experimental condition from the different experimental rounds. Per experimental condition, the data were represented by at least 3 chips, each containing at least 7 ROIs. The data in the results and figure legends are reported as mean ± SD, N corresponds to the number of ROIs. In the directionality analysis, ROIs with a fit of the graph less than 0.2 were dismissed resulting in 29 Fc, 106 Jag1 and 58 Dll4 ROIs analyzed. Both the Efficiency of controlled sprouting and the Directionality results were analyzed with a Kruskal-Wallis test and a Dunn’s multiple comparisons test as normal distributions were not assumed.

qPCR was performed was performed in 3 separate replications, with 3 or 4 wells per condition (Fc, Jag1, Dll4), per replication (averaged for statistical analysis). These results were analyzed by repeated measures ANOVA (to take into account different baseline between separate replications), and by Tukey’s post hoc test.

## Data Availability

Section 1 Data Fluorescence microscopy, image analysis and quantitative PCR data have been deposited at 4TU.ResearchData and are publicly available as of the date of publication. DOIs are listed in the key resources table.
Section 2 Code All original code has been deposited at 4TU.ResearchData and is publicly available as of the date of publication. DOIs are listed in the key resources table.
Section 3 Any additional information required to reanalyze the data reported in this paper is available form the lead contact upon request.

## Footnotes

Supplemental information can be found online at https://doi.org/10.1016/j.isci.2022.104306.
