# Supplementary material for: Engineered patterns of Notch ligands Jag1 and Dll4 elicit differential spatial control of endothelial sprouting
Source: iScience. 2022 Apr 27;25(5):104306. doi: 10.1016/j.isci.2022.104306 (PMC9114529; doi:10.1016/j.isci.2022.104306)
Supplement: Document S1. Figure S1 and Table S1 [file mmc1.pdf]

iScience, Volume *25*

Supplemental information

Engineered patterns of Notch ligands Jag1 and Dll4 elicit differential spatial control of endothelial sprouting

Laura A. Tiemeijer, Tommaso Ristori, Oscar M.J. A. Stassen, Jaakko J. Ahlberg, Jonne J.J. de Bijl, Christopher S. Chen, Katie Bentley, Carlijn V.C. Bouten, and Cecilia M. Sahlgren

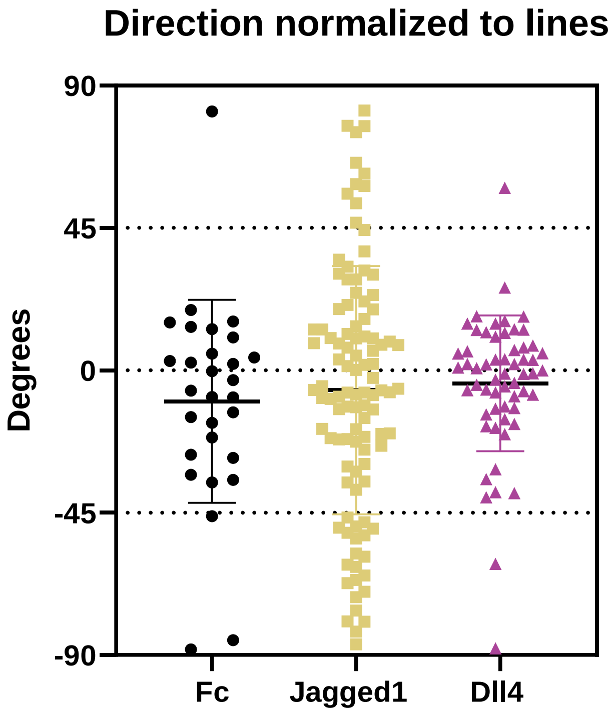

Figure S1. Directions of normalized lines, related to Figure 3
The average direction for every ROI, normalized to the direction of the lines (0 degrees). There is no significant difference between the mean direction between the sample groups. Data represented as Mean ± SD, N=29/106/58 ROI for Fc/Jag1/Dll4 respectively. Data points that represented ROIs with a “goodness of a fit” value lower than 0.2 were excluded.

Table S1. Parameter values, related to STAR Methods.

Parameter	Value	Description
$\boldsymbol{N_0}$	$1200\ molec\ \cdot h^{-1}$	Notch1 baseline production
$\boldsymbol{D_0, V_{R0}}$	$1000\ molec\ \cdot h^{-1}$	Dll4 and VEGFR baseline production
$\boldsymbol{J_0}$	$800\ molec\ \cdot h^{-1}$	Jag1 baseline production
$\boldsymbol{I_0, V_0}$	$200\ molec$	reference value of Notch and VEGFR activation
$\boldsymbol{V_{ext}}$	$1000\ molec$	external VEGF present in the cell environment
$\boldsymbol{\gamma}$	$0.1\ h^{-1}$	Notch protein degradation rate
$\boldsymbol{\gamma_S}$	$0.5\ h^{-1}$	activated Notch and VEGFR degradation rate
$\boldsymbol{k_T}$	$2.5 \cdot 10^{-5}\ h^{-1}$	transactivation rate
$\boldsymbol{k_C}$	$5 \cdot 10^{-4}\ h^{-1}$	cis-inhibition rate
$\boldsymbol{n_N, n_D, n_V, n_{V_R}}$	2.0	sensitivity of protein production to signaling activation
$\boldsymbol{n_J}$	5.0	sensitivity of Jag1 production to Notch1 activation
$\boldsymbol{n_F}$	1.0	sensitivity of Fringe production to Notch1 activation
$\boldsymbol{\lambda_{I,N}, \lambda_{I,J}, \lambda_{V,D}}$	2.0	effect of signaling activation on protein production
$\boldsymbol{\lambda_{I,D}, \lambda_{I,V_R}}$	0.0	effect of signaling activation on protein production
$\boldsymbol{\lambda_{F,D}}$	3.0	effect of Notch1 activation on Dll4-Notch1 binding as mediated by Fringe
$\boldsymbol{\lambda_{F,J}}$	0.3	effect of Notch1 activation on Jag1-Notch1 binding as mediated by Fringe
$\boldsymbol{D_{line}, J_{line}}$	$0 - 5000\ molec$	Dll4 and Jag1 proteins on the printed micropatterns
